# The Evolution of Public Sentiments During the COVID-19 Pandemic: Case Comparisons of India, Singapore, South Korea, the United Kingdom, and the United States

**DOI:** 10.2196/31473

**Published:** 2022-02-10

**Authors:** May O Lwin, Anita Sheldenkar, Jiahui Lu, Peter Johannes Schulz, Wonsun Shin, Chitra Panchapakesan, Raj Kumar Gupta, Yinping Yang

**Affiliations:** 1 Wee Kim Wee School of Communication and Information Nanyang Technological University Singapore Singapore; 2 School of New Media and Communication Tianjin University Tianjin China; 3 Institute of Communication and Health University of Lugano Lugano Switzerland; 4 School of Culture and Communication University of Melbourne Melbourne Australia; 5 Institute of High Performance Computing Agency for Science, Technology and Research Singapore Singapore

**Keywords:** COVID-19, public sentiment, Twitter, crisis communication, cross-country comparison, sentiment, social media, communication, public health, health information, emotion, perception, health literacy, information literacy, digital literacy, community health

## Abstract

**Background:**

Public sentiments are an important indicator of crisis response, with the need to balance exigency without adding to panic or projecting overconfidence. Given the rapid spread of the COVID-19 pandemic, governments have enacted various nationwide measures against the disease with social media platforms providing the previously unparalleled communication space for the global populations.

**Objective:**

This research aims to examine and provide a macro-level narrative of the evolution of public sentiments on social media at national levels, by comparing Twitter data from India, Singapore, South Korea, the United Kingdom, and the United States during the current pandemic.

**Methods:**

A total of 67,363,091 Twitter posts on COVID-19 from January 28, 2020, to April 28, 2021, were analyzed from the 5 countries with “wuhan,” “corona,” “nCov,” and “covid” as search keywords. Change in sentiments (“very negative,” “negative,” “neutral or mixed,” “positive,” “very positive”) were compared between countries in connection with disease milestones and public health directives.

**Results:**

Country-specific assessments show that negative sentiments were predominant across all 5 countries during the initial period of the global pandemic. However, positive sentiments encompassing hope, resilience, and support arose at differing intensities across the 5 countries, particularly in Asian countries. In the next stage of the pandemic, India, Singapore, and South Korea faced escalating waves of COVID-19 cases, resulting in negative sentiments, but positive sentiments appeared simultaneously. In contrast, although negative sentiments in the United Kingdom and the United States increased substantially after the declaration of a national public emergency, strong parallel positive sentiments were slow to surface.

**Conclusions:**

Our findings on sentiments across countries facing similar outbreak concerns suggest potential associations between government response actions both in terms of policy and communications, and public sentiment trends. Overall, a more concerted approach to government crisis communication appears to be associated with more stable and less volatile public sentiments over the evolution of the COVID-19 pandemic.

## Introduction

### Background

COVID-19 has infected people from more than 200 countries since it was first reported in late December 2019 [1]. Countries worldwide have put forth various precautionary measures at different time points in response to the rapidly evolving local disease situations [2,3]. With widespread global media coverage of the crisis and differing government approaches to COVID-19, it is important to understand public sentiments toward the pandemic in relation to governmental actions.

The proliferation of information and communications technology has widened the means for crisis communication since the beginning of the 21st century, particularly with the emergence and rapid propagation of the internet. Governments worldwide have used digital media to provide timely dissemination of information and education materials to a large population at low costs. For example, the widespread use of social media has facilitated crisis communication during recent disease outbreaks such as H7N9, Ebola, and Zika [4-6].

Public sentiment refers to the public’s opinion or attitude about a situation or something, which can be positive, negative, or neutral. By understanding the frequency of positive and negative public sentiments, policy makers and stakeholders can gain a clear picture of how people experience a given situation or policy and use such information to inform and calibrate how to more effectively communicate with the public to promote desirable behaviors and prevent negative behaviors [4]. The information gathered can also be used for future pandemic preparedness and crisis management.

In the era of social media, the evolution of public sentiments during the COVID-19 pandemic are highly complex and need to be empirically determined [7]. For example, discourse on social media can intensify negative public sentiments because much of what is propagated there is exaggerated, such as the potential threats of the disease [8]. Online fake news and biased comments are also circulated with ease [5,9], biasing public sentiments toward the disease. Moreover, COVID-19 is a fast-spreading disease that is harder to control than normal influenza because transmission can occur before symptom onset [10]. Thus, government communication on COVID-19 may become less effective in containing negative public sentiments, which can create potential situations of public panic that increase negative behaviors such as panic buying, hoarding, and violent political protest.

Several studies have examined public sentiments surrounding COVID-19 on social media for specific countries [11,12] and worldwide [13]. However, exploring the sentiment difference across multiple countries that have put different national measures in place is important for understanding the perceived public sentiments toward the effectiveness of these measures at macro levels. To the best of our knowledge, no study to date has examined the differences in public sentiments across multiple countries, over a longitudinal trajectory of the pandemic. Examining the differences across geographic locations and the trajectory of public sentiment changes is likely to reveal more dynamic insights than simply examining the frequency of positive and negative sentiments for a given specific time point or for a specific country.

This study attempts to close the knowledge gap by examining how positive and negative sentiments surfaced on Twitter in 5 countries since the early phase of the COVID-19 pandemic over 16 months. We purposefully compare data from 5 countries, namely, India, Singapore, South Korea, the United Kingdom, and the United States. The reason for selecting these countries were the existence of a substantial threat and diversity. The magnitude of the threat is detailed in the following section. Diversity concerns not only geographical and cultural diversity but also different disease trajectories and, linked to that, different and changing government stances on the best way to contain the virus. Diversity also referred to different attention to the countries with regard to the COVID-19 situation. Although the share of Twitter users vary within these countries (Singapore 13.6%, South Korea 22.8%, India 3.7%, the United States 10.3%, the United Kingdom 15.2%), they can still provide a snapshot of the discourse surrounding COVID-19 within a diverse group of situations [14]. This research does not include certain countries that were also highly impacted by COVID-19. For example, China, where the disease outbreak began, was omitted, as they have blocked Twitter and use other local social media platforms such as Weibo [15]. We describe the detailed background of the COVID-19 epidemics in the 5 countries in the next section to further elaborate our rationales for the selection of countries.

### COVID-19 in the 5 Countries

The World Health Organization (WHO) declared the disease outbreak as a Public Health Emergency of International Concern (PHEIC) on January 30, 2020, and its risk was upgraded to a “very high” global level on February 28 [16]. Two weeks later, on March 11, the WHO made the assessment that COVID-19 could be characterized as a “pandemic” [17].

**Figure 1:**
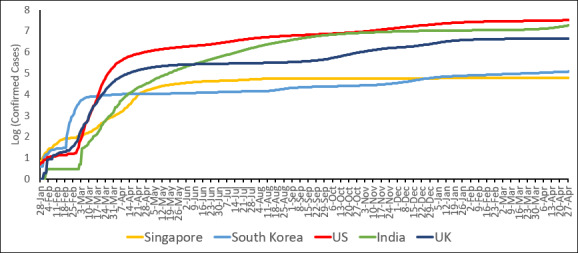
Logged numbers of COVID-19 cases in Singapore, South Korea, India, the United Kingdom, and the United States.

The trajectories of COVID-19 in the 5 selected countries demonstrated good diversity (Figure 1). For each country, we show the daily case numbers in the logarithmic function with a base of 10 to clearly present the trend of confirmed cases. The key events are also highlighted in Multimedia Appendix 1. Figure 1 shows that Singapore, as an Asian travel hub, was one of the first countries outside China to face the new threat. The local spread was well controlled throughout February and early March 2020, due to various containment measures. However, the country had an accelerated increase in the number of cases in mid-March due to the upsurge of imported cases and the outbreak in migrant worker dormitories [18]. The number of cases peaked in April and has since had a steady rate of a relatively low number of cases.

In comparison, South Korea witnessed a sudden spread of the disease throughout February until March 10, 2020, after several national measures were implemented to combat the disease, resulting in a plateau [3]. The number of cases remained stable until mid-August when cases began to rise again following another wave of the disease. A third wave also occurred in November 2020. Though numbers remain relatively low compared to other countries worldwide, COVID-19 cases in South Korea surpassed Singapore in late December 2020 [19].

India only had a few confirmed COVID-19 cases until March 2020 when the daily number rapidly increased. The cases remained at a high level and peaked on September 19, 2020. The number of daily cases reduced toward the end of the year and remained relatively constant until early March 2021 when a new wave of a more potent variant of the disease started to spread [20]. As of May 2021, India has become the new epicenter of COVID-19 and has surpassed the United States for the highest number of recorded cases in a day on April 22, 2021 [21].

The number of confirmed cases remained low in the United Kingdom until March 2020 when cases started to rise. The number of daily cases peaked in late April 2020 before falling throughout May and June 2020. It remained relatively steady until September 2020 when the number of cases increased again, surpassing the previous peak in April. The cases currently remain high but stable.

The United States saw an exponential increase in the number of confirmed cases in March 2020, quickly becoming the global epicenter of the disease and surpassing other countries to become the country with the highest number of cases in the world [22]. The number of cases peaked in January 2021, before falling in February 2021. The number of new cases remains steady but relatively high.

In response to the pandemic, the 5 countries have also used diverse strategies in crisis responses and public health communication, the details of which can be found in Multimedia Appendix 1. Singapore and South Korea took unique paths but were similar in terms of decisive actions and regular communication from the governments since their early phases of the epidemic. Both countries enforced some of the toughest measures early on, such as a national lockdown, widespread testing, and extensive contact tracing [3]. Health authorities communicated to the public regularly to address the outbreak, provide advice on preventative measures such as personal hygiene and social distancing, and announce disciplinary actions for people who do not follow the mandatory policies in place [23,24]. However, though Singapore’s response continues to receive praise by citizens as cases continue to be low and stable, several waves of cases in South Korea have led to the public criticizing the government for mixed messaging and caused unrest in health care workers [25]. Although the vaccination rollout has been steady and timely in Singapore, the South Korean government has faced backlash from the public for their slow rollout actions [26].

Despite having few cases in early 2020, India implemented a series of COVID-19 regulations early on with travel restrictions, quarantine, and a full lockdown when the number of cases started to rise in March 2020. However, the country was criticized for its lack of COVID-19 testing and delay in providing social support for residents upon enforcing lockdown. Although individual states had varying responses to the pandemic, the number of cases and death rates remained relatively low throughout 2020, and India’s strategy garnered praise from its citizens and other countries [27]. This led to an easing of measures, with the allowance of mass gatherings and politicians claiming the country had *beaten the pandemic* [28]. In early 2021, India was also praised for their proactive step toward providing free vaccines to citizens [29]. However, mid-March 2021 saw a second, more virulent wave, leading many to criticize the government’s response to the disease [30].

The United Kingdom has seen varying approaches by its constituent countries (England, Scotland, Northern Ireland, and Wales) and has been criticized for its contradictory and indecisive regulations [31,32]. The country delayed its response to the pandemic in March 2020. With the increase of cases, the country went into lockdown at the end of March for which the government was slated due to the late response. As cases reduced, regulations were loosened, leading to an increase in cases. The government responded with local and tiered restrictions, which were criticized for being complicated and confusing. With a new strain of COVID-19 appearing in the United Kingdom at the end of 2020, the country implemented several restrictions and regional lockdowns to stem further spread of the disease [33]. The lack of forewarning so close to Christmas caused a backlash among the public [34]. The United Kingdom was the first country in the world to initiate a vaccination program in December 2020 with the Pfizer vaccine, and to date, it has the second-highest vaccination rate in the world [35].

Similar to India, the United States has been less centralized in its approach, with many individual states varying in their actions [36,37]. The first case was discovered in late January 2020, and the national response was to reassure the public by downplaying the disease severity. Testing and diagnosis of the disease were slow due to barriers from the US Centers for Disease Control and Prevention (CDC) and Food and Drug Administration [38]. With increasing cases, the government suggested social distancing as a preventative measure. On March 13, 2020, after a substantial increase in cases, the United States declared the pandemic a national emergency, and more states began to implement stay-at-home notices, with differing directives being metered out [39]. Over the coming months, the country was criticized for its mixed and often contradictory messages from health authorities and the president [40,41]. Rather than enforcing countrywide mandates, governors were given a choice to control preventative measures within each state at a county level, leading to varying control measures across the country [42]. With the number of cases remaining stable but still relatively high, many states began to reopen in the summer months, causing a further increase in cases occurring toward the end of 2020, peaking in January 2021. The administering of the COVID-19 vaccines in early 2021 saw a decline in the number of cases, with over 100 million vaccines being administered by March 19, 2021, though mixed messaging and the antivaccination movement has led to varying rates of vaccination among the different states [43].

### Study Focus

With varying key events, regulations, and case numbers within the 5 countries, this paper examines how negative and positive sentiments evolved over the first 16 months of the pandemic for each country. By identifying how events and government crisis response within the pandemic have affected the public perceptions of disease threat across countries, we aim to provide critical case insights for policy makers to create effective response strategies to ensure more stable public sentiments.

## Methods

### Data Source

The study was approved by the Nanyang Technological University Institutional Review Board IR-2020-02-31 and was also reviewed and approved as “Exemption from full A*STAR IRB Review” (institutional review board reference number 2020-258). We used the COVID-19 Twitter Dataset with Latent Topics, Sentiments and Emotions Attributes [44] for our analysis. This data set was collected from Twitter’s standard search application programming interface (API) using 4 COVID-19–related search words—“wuhan” (which at the start of the pandemic was commonly used in relation to the virus), “corona,” “nCov,” and “covid,”—in the English language. For each retrieved record, the API returns a tweet ID, tweet text content, timestamp, a user ID, and a location that is part of the tweet author’s public profile, among other attributes. As the “location” attribute is an open-ended field that can contain both geographically meaningful information (eg, “Ontario, Canada” or “London) or otherwise (eg, “online” or “The Entire Universe!”), the country mapping was obtained by having each “location” mapped with a country code using GeoNames cities15000 database [45]. According to Gupta et al [44], the data set has approximately 54% of the collected COVID-19–related tweets associated with meaningful country-identifiable “location” information.

For this study, our analysis comprised 7,814,109 country-identifiable tweets from India; 293,331 from Singapore; 68,903 from South Korea; 12,248,379 from the United Kingdom; and 46,938,369 from the United States. That is, we analyzed a total of over 67,363,091 Twitter posts focusing on the 5 countries of interest covering the 15-month period from January 28, 2020, to April 28, 2021.

In addition to the Twitter data set, for each country, we also collected the key events from the government and health authorities, and plot these events on the pandemic timeline. The composite of Twitter data is then set against the tweet data in each of the countries for detailed analyses.

### Data Processing, Sentiment Classification, and Analysis

The Twitter data were analyzed with an advanced sentiment analytic algorithm, *CrystalFeel*, which has been demonstrated to achieve state-of-the-art measurement accuracy [46] and is available as a complimentary web-based API service for research use [47]. The algorithm was trained and validated using features derived from both pretrained language models, word embedding, and an original handcrafted lexicon. This approach is superior as compared to a traditional bag-of-words approach, which does not have the inherent ability to correctly analyze sentiments from expressions that may or may not contain emotional words per se (eg, “What to do with my life...I have no more choices...”), or expressions with positive/negative words but the sentence-level sentiment is different (eg, “Arrrhhh I hardly feel *happy* any more these day...” or “He *cried* when he heard that his son had been found alive and well”). According to the evaluation study performed [46], CrystalFeel’s valence intensity achieved a very high measurement accuracy of 0.816 in terms of Pearson correlation coefficient (*r*) with manually annotated test data provided by a shared task on “affective in tweets,” organized at the SemEval 2018–international workshop on semantic evaluation [48]. CrystalFeel’s predictive validity was also tested and proven in other natural language processing tasks [49-52].

For a given text message (in this case, a tweet), the CrystalFeel API produces a sentiment score that indicates the intensity of the valence expressed in the text, where the valence intensity score corresponds to the degree of overall unpleasantness and pleasantness in the text expression, ranging from 0 (the text expresses extremely negative feelings) to 1 (the text expresses extremely positive feelings). For this study, we used CrystalFeel API service’s sentiment labels converted from valence intensity scores for more straightforward interpretation [47], namely, “very negative” (valence intensity ≤0.30), “negative” (valence intensity 0.30-0.48), “neutral or mixed” (valence intensity 0.48-0.52), “positive” (valence intensity 0.52-0.70), and “very positive” (valence intensity ≥0.70).

Based on the sentiment labels, the data for our analysis were then aggregated as the count or volume of “very negative,” “negative,” “neutral or mixed,” “positive,” and “very positive” tweets collected for each day.

In addition, as each country has different levels of total tweet volumes, we computed a normalized “positivity” score for each country every day to facilitate cross-country comparisons and understand whether there had been more positive or negative sentiments in each country. This positivity score, expressed as the following formula, was calculated as the difference in the number of positive and negative tweets on a day over the total number of tweets of each of the 5 countries.

Positivity = [(Number of very positive + positive tweets) – (Number of very negative + negative tweets)] / Total number of tweets per day

The higher the normalized positivity scores, the higher the volumes of positive tweets in the discourse. A low score indicates an overwhelming volume of negative public sentiments. A score of zero would indicate an exact balance between positive and negative sentiments.

## Results

### Sentiment Trends in Relation to Key Disease Events and Government Responses

In the following section, we describe the volume of tweets and the normalized positivity score with different qualitative labels of sentiments by each country and key global and local responses. Overall, negative sentiments were expectedly predominant across all countries, especially after the WHO’s pandemic declaration on March 11, 2020. Positive sentiments also surfaced in each country after the declaration, more so in Asia than in the west, although to a relatively lesser extent, with “very positive” sentiments being scarce.

### Singapore

**Figure 2:**
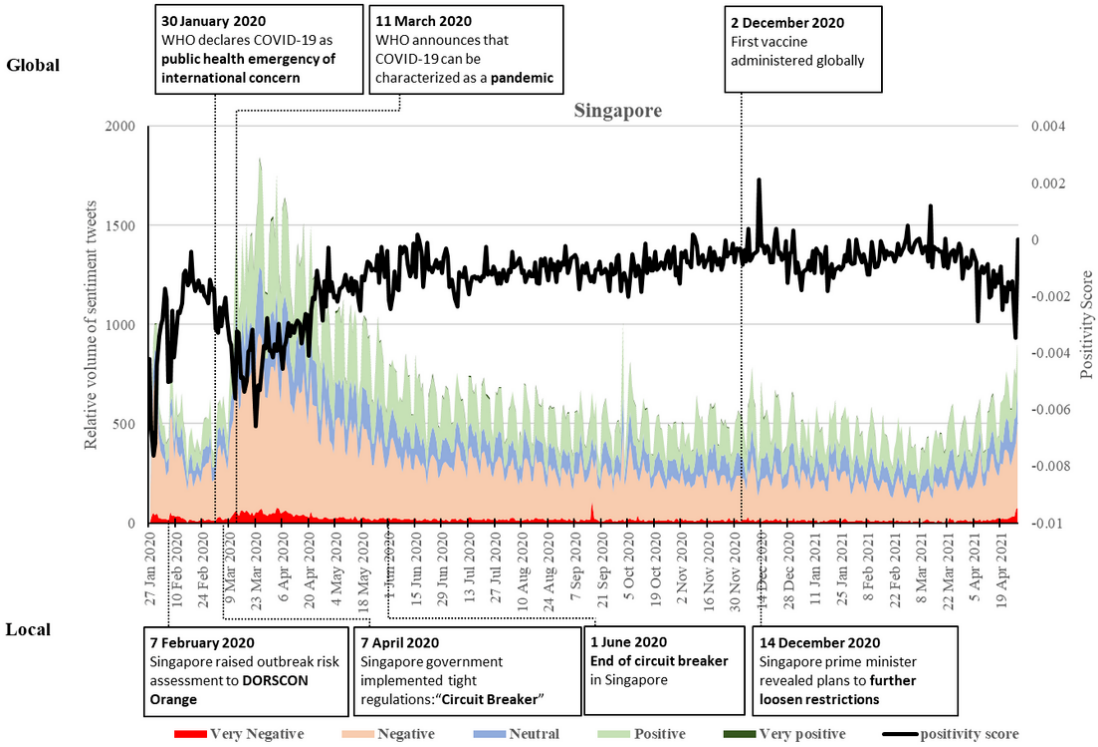
Twitter sentiments in Singapore from January 28, 2020, to April 28, 2021. DORSCON: Disease Outbreak Response System Condition; WHO: World Health Organization.

On January 30, 2020, when the WHO declared the disease outbreak as a PHEIC, Singapore witnessed a significant Twitter proliferation of negative sentiments, leading to a low score of positivity at the beginning of the pandemic (Figure 2). There had been several confirmed cases and the declaration heightened the threat of local spread. The frequency of negative tweets decreased in the next week but increased again on February 7 when Singapore raised the outbreak risk assessment to Disease Outbreak Response System Condition (DORSCON) “Orange,” meaning the disease was “severe and spread easily, but still contained” [53]. The DORSCON announcement resulted in a balanced sentiment in posts. After that, the negative sentiments were relatively low for a month, corresponding to the containment efforts of local disease spread during this period. Both negative and positive tweets increased after the WHO declaration of COVID-19 as a pandemic, with the positivity score decreasing.

The volume of sentiments peaked after the categorization of COVID-19 as a pandemic and reached its highest level on March 25, 2020, witnessing the second largest dip of the positive score on this day. This concurred with the rapid growth of confirmed cases due to the worsening situation worldwide and tighter measures being implemented in the country, including safe distancing policies requiring at least 1 meter between persons. Although there was a surge of infections in migrant workers living in dormitories, leading to the highest number of cases occurring on April 20, 2020, both negative and positive sentiments decreased in April though they remained relatively high. During this time the government implemented tight regulations such as a “Circuit Breaker” on April 7, requiring citizens to stay at home except for essential trips [18]. They provided regular updates on the number of cases and the methods taken to reduce further spread. The daily volume of sentiments continued to decrease through May and the end of Circuit Breaker on June 1. Thereafter, the overall positivity of tweets remained stable from April 2020 to the end of March 2021. However, there was a spike in the volume of both positive and negative sentiments in October 2020 when the outbreaks at the dormitories finally abated. The highest positivity was witnessed on December 14, 2020, when the Prime Minister addressed the COVID-19 situation and revealed plans to enter “Phase 3” of the pandemic with further loosening of the restrictions due to the low number of cases.

Since March 2021, there has been an increase in negative sentiments, and the positivity score is still on a downward trajectory. This reflects the number of cases increasing and the news that many countries are affected by a third, more virulent wave of the disease.

### South Korea

**Figure 3:**
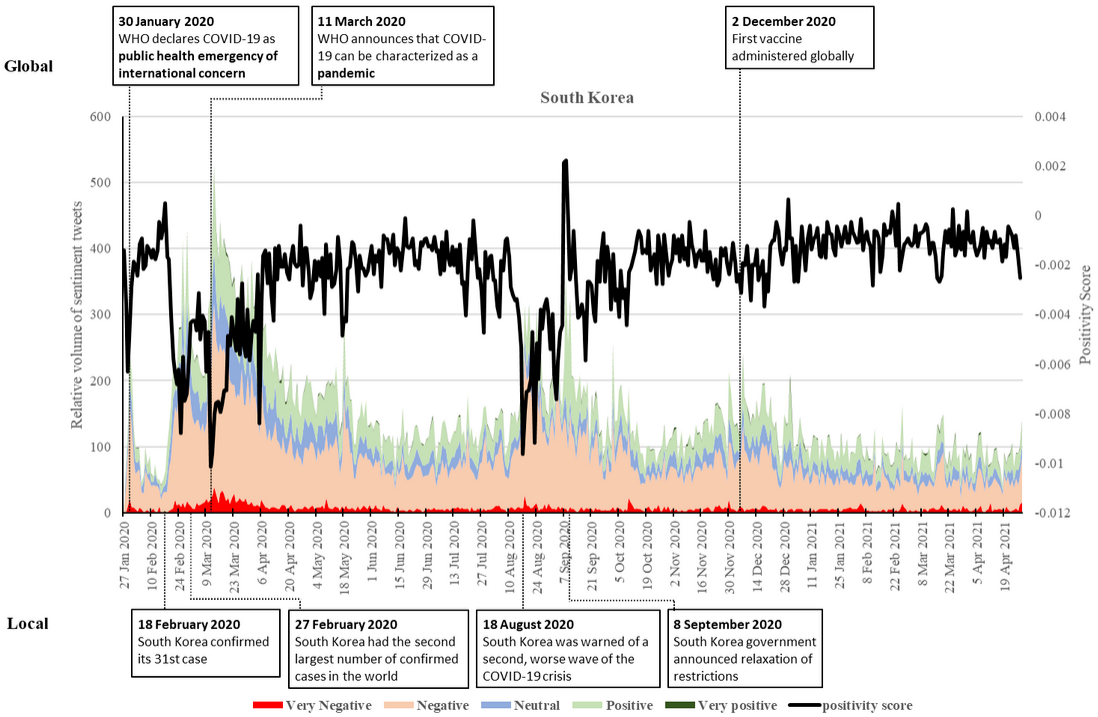
Twitter sentiments in South Korea from January 28, 2020, to April 28, 2021. WHO: World Health Organization.

Similar to Singapore, South Korea saw significant fluctuations in sentiments at the start of the pandemic (Figure 3). There was a substantial increase in negative sentiment on January 30, 2020. The frequency of negative tweets decreased in the next 20 days. However, it started increasing on February 18 when the country confirmed its 31st case, who was known as a member of a quasi-Christian cult “Shincheonji” and believed to pass the infection to a number of fellow worshipers at the church located in Daegu, the fourth largest city in South Korea. The number of confirmed cases in the country increased from 30 cases on February 17 to 100 on February 20, swiftly soared to 1000 on February 27, 2000 on February 28, and 3000 on February 29. The number of negative tweets saw its first peak at the end of February, and the negative sentiments overwhelmed the country within this short period. On February 27, South Korea had the second-largest number of confirmed cases in the world. Positive tweets remained at a relatively stable volume during this time with a minor increase during mid-February, when the country started to put control measures into place.

The frequency of negative tweets decreased in late February 2020 and early March, as the country began to implement various measures to fight COVID-19, including drive-through sample collection facilities, mobile phone alerts notifying people of new cases near them, and the “self-quarantine safety protection” app. This smartphone app keeps track of the locations of those who have been ordered not to leave home [54]. This measure is reflected in the increase of positivity in sentiments. The country carried out more than 200,000 tests as of March 11, 2020. The number of new confirmed cases has remained low since then. The number of tweets surged from March 11-13, 2020, after the WHO declared the COVID-19 outbreak a pandemic, with the negative posts roughly doubling the number of previous peaks and more positive sentiments surfacing. Nevertheless, negative comments gradually went down, while the positive sentiments remained high as the country began to flatten the curve, resulting in a relatively high positivity score in the next few months.

The negative sentiments surged again on August 18, 2020, when the country was warned of a second worse wave of the COVID-19 crisis spreading from Seoul churches. As the number of daily cases reduced and rules began to loosen, sentiments began to balance out. The positivity score increased substantially, with positive sentiments surpassing negative ones in early September, peaking on September 9, 2020, when the government announced a relaxation of restrictions on operations of cafés and bakeries. Concurring with the third wave of COVID-19 outbreak worldwide, South Korea witnessed a surge of negative sentiments in late November and early December, though to a lesser extent than the previous two waves. As the disease curve was flattened, the negative sentiments gradually decreased until the study period. As such, the positivity score remained relatively stable, although slightly more negative with tiny spikes in positivity.

### India

**Figure 4:**
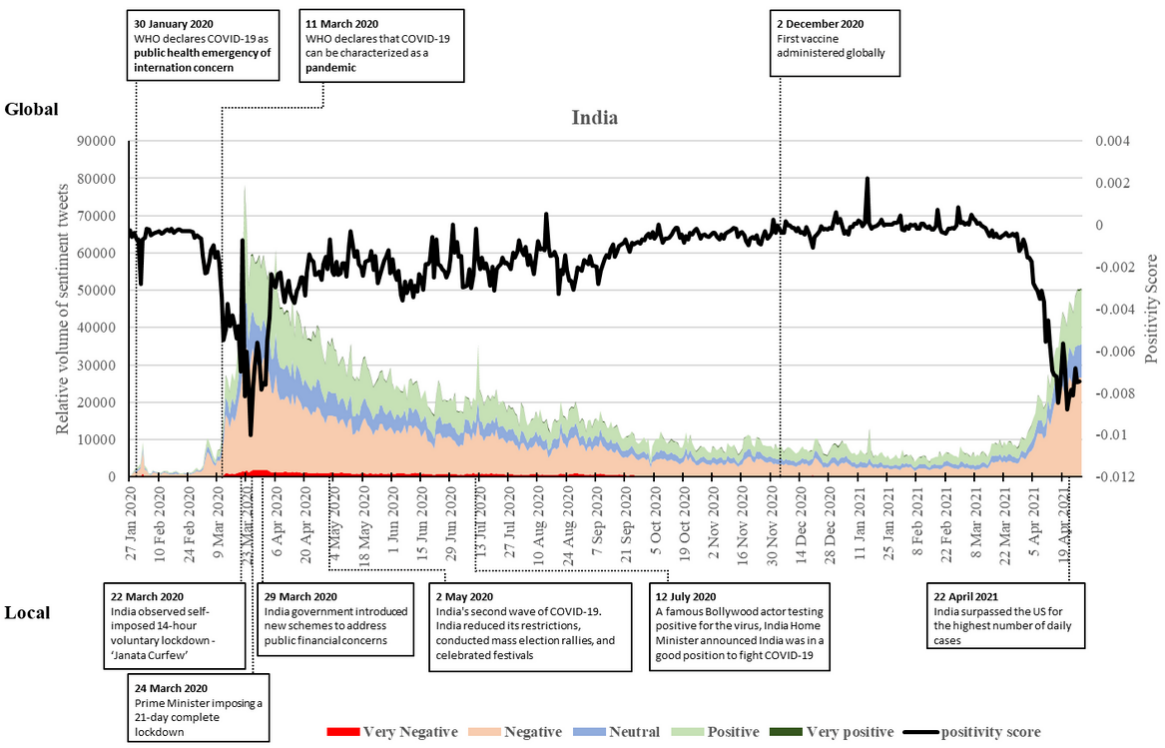
Twitter sentiments in India from January 28, 2020, to April 28, 2021. WHO: World Health Organization.

India saw relatively balanced sentiments at the start of the pandemic with a small spike of negative sentiments on February 2, 2020, with the second confirmed case and COVID-19 spreading worldwide (Figure 4). The number of negative posts remained relatively low until March 2020, echoed by the few reported cases within India during this period. After that, sentiments became overwhelmingly negatively skewed except for a spike in positivity on March 22, with the introduction of the “Janata Curfew” [55]. March 26, 2020, saw the lowest positivity score with the first day of the nationwide lockdown. There was an upward trend in positive posts on March 29, 2020, with the government’s introduction of rapid solutions such as new schemes and moratoriums on loan repayments to address public financial concerns [56].

After this peak, the volume of negative and positive tweets started decreasing, particularly the number of negative sentiments. The positivity score increased with sentiments becoming more balanced on April 6, 2020, and remained stable until August. A small spike in the volume of both positive and negative sentiments was seen on July 12, 2020, with the news of a famous Bollywood actor testing positive for the virus and the home minister announcing that India was in a good position to fight COVID-19 [57]. On August 15, 2020, sentiments became more positive than negative on Indian Independence Day. The volume of both negative and positive tweets continued to decrease, and sentiments remained balanced until December 2020, reflecting the decrease in daily COVID-19 cases. On December 31, 2020, there was a slight increase in positive tweets with the end-of-year celebrations.

This balance continued from early January to March 2021, with some small spikes toward more positive sentiments as India announced vaccine maitri (Vaccine friendship) to its neighboring countries [58]. The positivity score became highest on January 16, 2020, with overwhelmingly positive sentiments, as the prime minister launched the world’s largest vaccination drive. However, March 2021 saw a sudden downturn in the positivity score, as negative sentiments began to increase back to the March 2020 levels with the new, more deadly wave of cases. Negative tweets reached more than 20,000 by mid-April and peaked on April 27, 2021. India saw the second wave of COVID-19 with exponential increases in infections and death rates. As of May 2, 2021, India reported more than 300,000 cases per day, after the country reduced its restrictions, conducted mass election rallies, and celebrated festivals. As of this writing, sentiments are still highly negative, as the disease continues to affect India.

### United Kingdom

**Figure 5:**
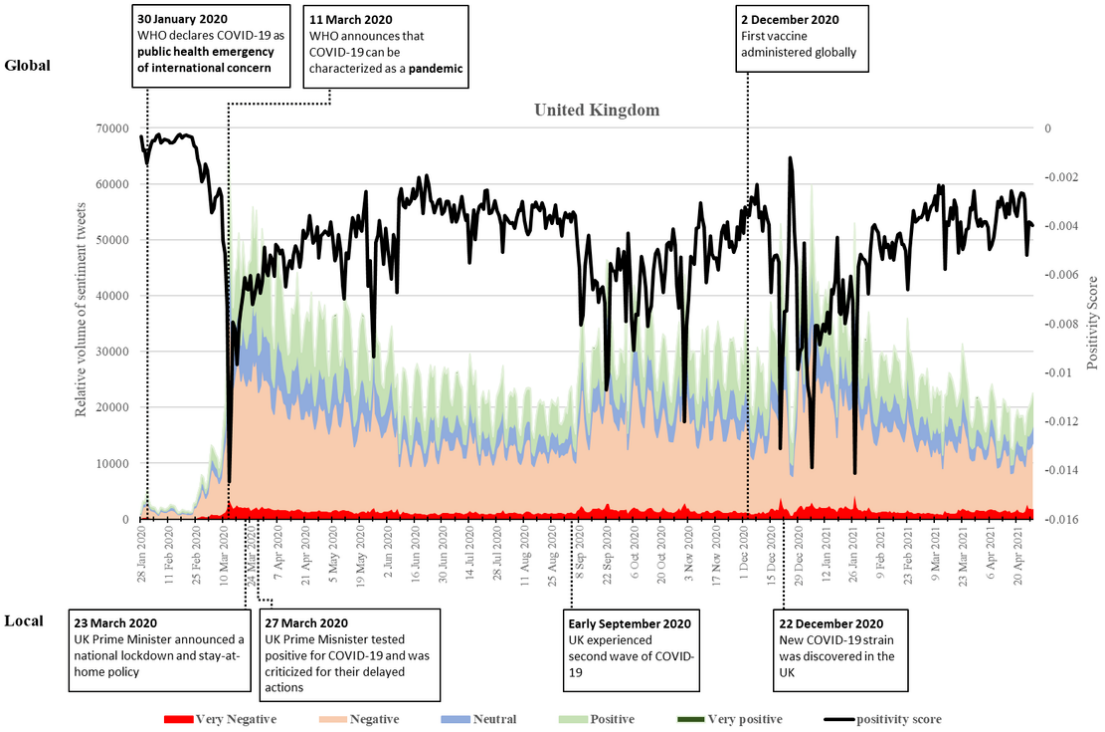
Twitter sentiments in the United Kingdom from January 28, 2020, to April 28, 2021. WHO: World Health Organization.

Unlike the Asian countries investigated, the United Kingdom saw only a minor spike of tweets when the WHO declared the COVID-19 as a PHEIC (Figure 5). The tweets began to surge only in late February and early March 2020 when more COVID-19 cases were confirmed. This resulted in little change of the positivity score until early March 2020. The most significant upsurge of the negative sentiments was on March 13, after the WHO’s declaration of the pandemic, and major events were canceled.

Positive sentiments surged quickly after the UK prime minister delivered a nationwide speech that encouraged the citizens’ efficacy in fighting the disease and announced a national lockdown and stay-at-home policy on March 23, 2020. Nevertheless, the surge of positive tweets lasted only for a week and then dipped on March 27 when the prime minister tested positive for the disease. The country was criticized for its delayed actions in preventing the spread of COVID-19 [31,32]. Though the positivity score soon recovered in April, it showed dips in late May and early June when the UK prime minister announced the loosening of the national lockdown while at the same time the country recorded more than 40,000 deaths due to the disease. The positivity score thereafter increased and remained relatively stable during the summertime.

Nevertheless, negative sentiments witnessed significant upsurges again in early September and late December 2020, when the second and the third waves of the disease hit the United Kingdom, resulting in fluctuating and negatively skewed sentiments. Particularly, tiered restrictions were introduced in the UK countries in October. In December, a new COVID-19 variant led to an increase in cases. The United Kingdom witnessed a large dip on December 20 when the prime minister declared, “We cannot continue with Christmas as planned,” requiring residents to stay at home during the Christmas holidays [34]. However, a few days later, on December 26, 2020, there was a sudden increase in the positivity score as new restrictions were introduced around the United Kingdom. On January 5 and January 27, 2021, negative tweets increased and reached similar levels early in the pandemic on March 13, 2020, as the prime minister gave statements regarding the COVID-19 situation. Since February 2021, the UK positivity score has increased, with more balanced sentiments as the number of cases remains relatively low and the vaccination has been extensively rolled out. The country has, as of this writing, vaccinated over half of its population. Overall, the United Kingdom did not skew toward positive sentiments during the study period.

### United States

**Figure 6:**
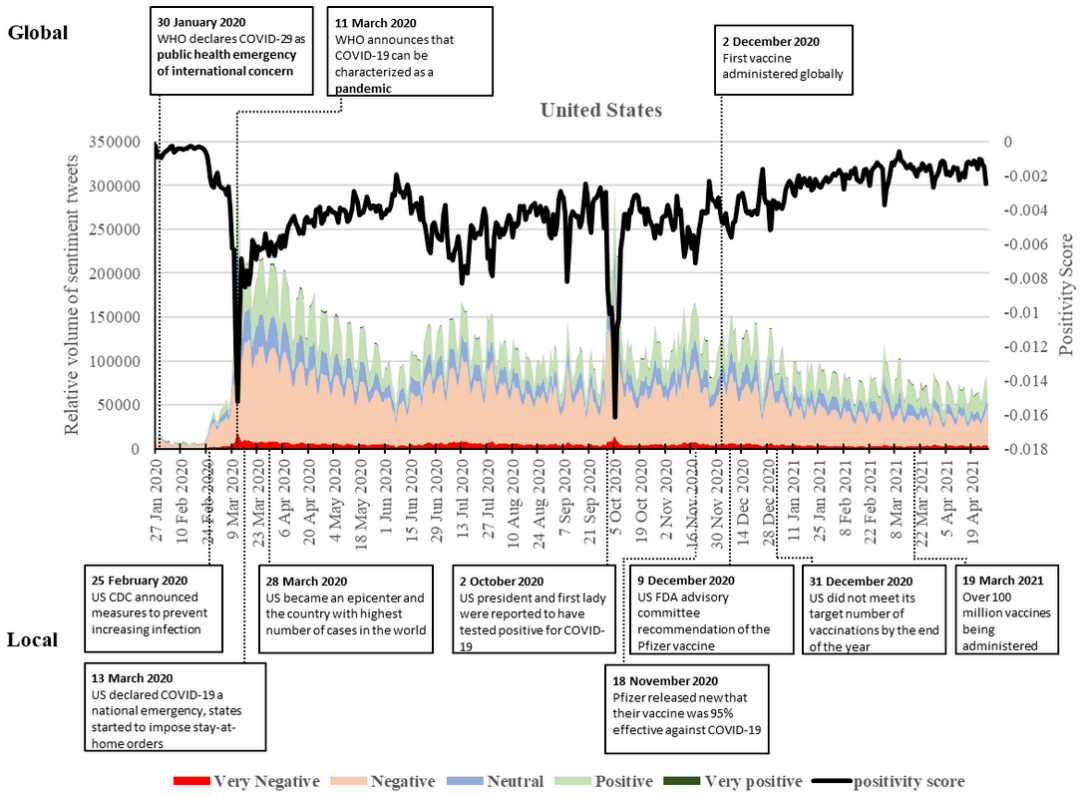
Twitter sentiments in the United States from January 28, 2020, to April 28, 2021. CDC: Centers for Disease Control and Prevention; FDA: Food and Drug Administration; WHO: World Health Organization.

The WHO’s declaration of COVID-19 as a PHEIC on January 30, 2020, led to a small spike in the relative volume of negative tweets in the United States, similar to the United Kingdom (Figure 6). The relative volume of both positive and negative tweets remained low until the last week of February. This resulted in little change in sentiments at the start of the pandemic. On February 25, the CDC announced the pandemic was likely to spread to the United States and measures should be put into place to prevent the infection rate from increasing. The announcement coincided with the first major increase of negative tweets. The WHO raised the threat level of the disease to “very high risk” on February 28, the day when the first peak of negative tweets occurs. Few positive tweets were seen at this time. During this period, the positive score gradually became more negative along with the increasing number of cases within the country.

The biggest increase in negative tweets, over 30 times more than the peak on January 30 and 5 times than that on February 28, occurred on March 12 and 13, 2020. This increase followed closely upon the announcement on March 11 by the WHO’s pandemic declaration and the US’s national declaration of emergency on March 13. Meanwhile, the positivity score saw a substantial decrease. However, the dip was much lower than those in the other countries, suggesting an overwhelming number of negative tweets on that day. The reassurance by the government also saw positive sentiment surfacing, leading to an increase in the positivity score.

The volume of both positive and negative sentiments gradually decreased from March 13 to early June 2020 when the number of cases reached 2 million and states started to impose stay-at-home orders. However, the positivity score remained at a negative level, fluctuating in negative sentiments around the summer of 2020 when there was a rise in COVID-19 cases. The number of positive and negative sentiments then began to rise again, with cases increasing rapidly and news of vaccine development and efficacy during trials showing positive results. This increase in tweets culminated in a smaller peak on July 15 as daily cases reached a new high.

Sentiments remained relatively high but stable until October 2, 2020, when negative sentiments rapidly increased as the president and the first lady were reported to have tested positive for COVID-19 [59]. The increase in negative sentiments peaked on October 6—with the president seen discharged from the hospital—and quickly fell back to levels found over the summer. A peak in positive sentiments was found on November 18 as Pfizer released news that their vaccine was 95% effective against COVID-19. However, there was also an increase in negative sentiments as the number of cases surpassed 11 million, and citizens were advised by the CDC to stay home for Thanksgiving. On December 9, 2020, though negative sentiments were still the majority, positive sentiments were found in the tweets with the US Food and Drug Administration advisory committee’s recommendation of the Pfizer vaccine, and the world’s first COVID-19 vaccine was administered to members of the public in the United Kingdom [35]. On December 22, a small peak in negative sentiments was found as a new strain was discovered in the United Kingdom [33]. On December 31, another slight increase in negative sentiments occurred as reports surfaced that the United States did not meet its target number of vaccinations by the end of the year [60]. Since then, the relative volume of negative tweets remains very high compared to January and February from the previous year.

Though positive sentiments also remain higher than earlier in the pandemic and the positivity score has increasingly become more balanced, the score did not skew toward more positive sentiments than negative within the study period.

## Discussion

### Principal Findings

This study set out to examine the evolution of COVID-19 sentiment trends and the balance of positive and negative public sentiments in 5 countries over the course of the COVID-19 pandemic. The sentiment trajectory of each country within the framework of government actions provides unique implications for considering when and how negative sentiments overwhelm positive sentiments and may cause unanticipated public reactions. The findings of our study present important implications for policy making, as they indicate public perceptions of the disease threat in connection with government health crisis responses, which in turn could lead to large scale public behavior effects.

Our findings clearly demonstrate that Singaporean and South Korean populations showed different perceptions of the disease at the beginning of the COVID-19 pandemic compared to those in the other 3 countries and were immediately active on social media in response to the WHO’s declaration of PHEIC in January 2020. This indicates that the 2 countries have been vigilant since the early outbreak, possibly due to perceived closer geographic distance from the initial epicenter (China), higher air travel between the affected countries, and perceived potential disease spread. In addition, both Singapore and South Korea were previously affected by the severe acute respiratory syndrome (SARS) in 2003. They had therefore implemented pandemic preparedness initiatives to improve outbreak preparedness and the rapid handling of novel diseases [23,24]. In contrast, the public in the United States, India, and the United Kingdom demonstrated fewer reactions to the early declaration, suggesting consistency with fewer active cases reported in these countries during early 2020.

Our sentiment analyses also demonstrate a clear contrast between the 2 western countries versus the 3 Asian countries. Over the 16 months, relatively more minor sentiment swings appeared in South Korea, Singapore, and India, but wide swings in negativity were observed in the United States and the United Kingdom. Indeed, the 3 countries in Asia faced escalating waves of cases, which increased expected negative sentiments. However, for all 3 Asian countries, substantial proportions of positive sentiments also surfaced in parallel, balancing the overall negativity of public sentiments. In the United States and the United Kingdom, although negative sentiments increased substantially after the cases began to increase, similar solid positive sentiments were slow to surface, indicating potential public alarm and possibly frustrations within the populations. This could be due to the relatively clearer and stricter regulations implemented by Singapore and Korea upon discovering cases within their nations [3]. Furthermore, although India was slower to act initially upon the WHO declaration, their case-fatality rate remained low throughout 2020, which may have bolstered public positivity toward the pandemic response [27]. In both the United States and the United Kingdom, the initial lack of clarity of COVID-19 responses, along with mixed messaging and contradictory policies appear to have led to a much greater distribution of negative viewpoints from the public over the first 16 months of the disease timeline.

As the pandemic evolved, national-level government crisis responses and local disease developments appear to be strongly associated with the trends and fluctuations of public sentiments in all 5 nations. At a macro level, our findings demonstrate the correspondence between public sentiments and government actions. Overall, negative sentiments surged when local disease threats escalated and with local emergency measures such as the announcement of lockdowns. Conversely, the positive sentiments also increased in line with the government-initiated crisis responses like financial support and vaccination rollout. However, indecisive and contradictory crisis responses, such as those in the United Kingdom and the United States during their early epidemics, seemed to do more harm than good for the public’s positive sentiments. Additionally, infections of high-ranking government officials and celebrities induced negative sentiments consistently across countries, possibly because such incidents could amplify the perceived risks and reduce public trust in the government’s responses. This implies that governments need to provide initial timely responses to ease the public from the emergent threat during public health crises. Meanwhile, the authorities should also assure the public by maintaining a good impression and considering themselves as role models for the public.

Our findings also suggest that social media play significant roles in public health crisis responses. Overall, echoing previous studies [5-7], this study shows that social media sentiments are sensitive to both global and local crisis milestones. The public’s sharing of emotions through social media is an organically developed data source that shows the collective sentiments of the people. The shared positive and negative opinions can reflect the information or situation they face at a point in time. This up-to-date data is a valuable tool to evaluate and understand the emotional well-being of the public, their concerns regarding the new changes, new policy announcements, and the ongoing pandemic itself. This suggests that social media are important data sources for comparisons of local government responses during global public health crises and should be explored further.

### Limitations and Future Research

This research has a number of limitations that warrant future research. First, although our findings clearly showed that the trends of general positive and negative sentiments, and their differences, coincided with government decisions in fighting the disease, our focus is on positive and negative sentiment valences instead of discrete emotions. This choice gives us the advantage of clearly identifying the key differences and trend of change of the focal sentiment construct over a longitudinal scale of 16 months of data and across multiple countries of our analytical interest. In future work, it may be worthwhile to examine more specific shared emotional experiences such as the public’s collective fear, anger, happiness, and sadness [46,61,62], and their respective emotion frequency, intensity, and change over time following government measures and communications [7].

Second, the study retrieved and examined tweets in English. English is only one of the common languages in Singapore and India, and not widely used in South Korea. Therefore, information on public sentiments in these 3 Asian countries may not be fully captured by our data universe, which is an issue that is faced by many multicountry studies. The data obtained can be used as a general guide with the knowledge that it represents a subset of the population’s social media discourse. Nevertheless, future studies should investigate public sentiments with the inclusion of analysis from a broader range of local languages used in each country.

Third, it is useful to note that according to Gupta et al [44], approximately 54.2% of 198,378,184 tweets collected on COVID-19–related keywords have country-identifiable “location” information as declared by the users at their Twitter public profile. Although this is a reasonable representation, we would like to caution toward generalizing the data we used to fully represent the social media population for each country studied in this research.

Fourth, we used Twitter as a proxy for public sentiments on social media. Although Twitter has a high user base in countries such as the United States, India, and the United Kingdom [63], it is only one of many social media platforms and may have less frequent users for other countries, which could lead to selection bias. Future studies should consider expanding the range of platforms used to capture a broader range of social media, such as Reddit and Facebook, and explore the number of unique users posting to capture a wider range of sentiments.

Though these findings demonstrate the association between government and health authorities’ crisis responses and evolvement of public sentiments, future research needs to continue across the entire span of the COVID-19 course to attain a fuller understanding of the phenomenon and additionally use more in-depth qualitative methods, including case studies, to further scrutinize the linkages and examine the underlying mechanisms. In addition, specific discourses of public sentiments should be examined to reveal specific public opinion and social media responses toward the government acts and policies.

### Conclusion

This research is an initial attempt to compare long-term public sentiments in different countries, aiming to consider and guide policy implications to manage the unprecedented COVID-19 pandemic and future crises of similar nature. Our findings from longitudinal data over the first 16 months of the COVID-19 trajectory show that India, Singapore, and South Korea have seen relatively stable negative sentiments along with sizable positive sentiments. In contrast, the United States and the United Kingdom witnessed a substantial upsurge of negative sentiments, and parallel positive sentiments were slow to surface. Thus, it appears that concerted early responses to the pandemic are associated with overall positivity reflected in public sentiments. The research findings also suggest that more rigorous and consistent approaches of government crisis communications appear to be associated with more stable and balanced sets of public sentiments during the COVID-19 pandemic.

## Appendix

Multimedia Appendix 1 Key COVID-19 events by country between January 28, 2020, and April 28, 2021.

## Abbreviations

API: application programming interface
CDC: Centers for Disease Control and Prevention
DORSCON: Disease Outbreak Response System Condition
PHEIC: Public Health Emergency of International Concern
SARS: severe acute respiratory syndrome
WHO: World Health Organization
