# Supplementary material for: The Evolution of Public Sentiments During the COVID-19 Pandemic: Case Comparisons of India, Singapore, South Korea, the United Kingdom, and the United States
Source: JMIR Infodemiology. 2022 Feb 10;2(1):e31473. doi: 10.2196/31473 (PMC9987195; doi:10.2196/31473)
Supplement: Multimedia Appendix 1 [file infodemiology_v2i1e31473_app1.docx]

**Multimedia Appendix 1: Key COVID-19 events by country between January 28, 2020, and April 28, 2021**

| **Country** | **Date** | **Key Events by Country** |
| --- | --- | --- |
| Global events | 30 Jan 20 | WHO declares COVID-19 a “Public Health Emergency of International Concern |
|  | 28 Feb 20 | WHO upgrades the COVID-19 risk to “very high” |
|  | 11 Mar 20 | COVID-19 is characterized as a “pandemic” |
| Singapore | 23 Jan 20 | First confirmed case in Singapore |
|  | 7 Feb 20 | Singapore raises outbreak risk assessment to DORSCON Orange |
|  | 7 Apr 20 | Government implement lockdown “circuit breaker” |
|  | 20 Apr 20 | Surge of cases in migrant worker dormitories |
|  | 1 Jun 20 | End of circuit breaker |
|  | Oct 20 | cases in migrant dormitories abate |
|  | 14 Dec 20 | PM reveals plans for “phase 3“loosening of restrictions. Pfizer Vaccine approved for use in Singapore |
|  | Mar 21 | Number of cases rise |
| South Korea | 18 Feb 20 | Super-spreader case 31 confirmed, measures (e.g. isolating at home and testing travellers from China introduced) |
|  | 27 Feb 20 | South Korea has the second largest number of cases in the world |
|  | Late Feb 20 | Number of measures introduced (e.g. isolating in treatment centres and quarantine of Daegu where cases are high) |
|  | 23 Mar 20 | Strict social distancing is implemented such as no unnecessary travel outside of home |
|  | Apr 20 | Number of cases reduce |
|  | 18 Aug 20 | Country warned of second wave |
|  | 9 Sep 20 | Government announces relaxation of regulations |
|  | Late Nov 20 | third wave of covid-19 outbreak globally |
|  | 10 Feb 21 | Approval of Astra Zeneca Vaccine |
|  | 2 Feb 20 | Early community spread |
| India | 22 Mar 20 | Janata curfew implemented |
|  | 26 Mar 20 | First day of nationwide lockdown |
|  | 12 Jul 20 | Bollywood actor testes positive, Home Minister announces India in a good position to fight COVID-19 |
|  | Nov 20 | cases remain low |
|  | 16 Jan 21 | PM launches vaccination drive |
|  | Mar 21 | Cases rise exponentially |
|  | 22 Apr 21 | Surpasses US for highest number of cases in a day |
|  | 12 Mar 20 | Major events are cancelled |
| United Kingdom | 23 Mar 20 | PM announces national lockdown |
|  | 27 Mar 20 | PM tests positive for COVID-19. Country criticized for delays in action |
|  | Jun 20 | PM announces loosening of national lockdown while number of deaths remains high |
|  | Sep-Oct 20 | second wave hits UK and tiered restrictions are implemented |
|  | 5 Nov 20 | Second national lockdown in England |
|  | 8 Dec 20 | First COVID-19 vaccine administered to members of the public |
|  | 22 Dec 20 | Third wave hits UK with new variant discovered |
|  | 13 Mar 21 | PM gives speech on situation |
|  | Feb 21 | Cases remain low and vaccine rolled out extensively |
|  | 25 Feb 20 | CDC suggested measures should be put in place to prevent COVID-19 |
| United States of America | 13 Mar 20 | US declares COVID-19 a national emergency |
|  | Jun 20 | Cases reach 2milion and states start to impose stay at home orders |
|  | 2 Oct 20 | President and first lady tested positive for COVID-19 |
|  | 6 Oct 20 | President discharged from hospital |
|  | 18 Nov 20 | Pfizer releases news that vaccine is 95% effective against COVID-19 |
|  | Late Nov 20 | Citizens advised to stay home for thanksgiving by CDC |
|  | 9 Dec 20 | FDA recommends use of Pfizer |
|  | 19 Mar 21 | 100 million vaccine administered but anti vaccination movement is rising |
